# Implementation of an Automated Cerebrospinal Fluid Drainage System for Early Mobilization in Neurosurgical Patients

**DOI:** 10.3390/brainsci11060683

**Published:** 2021-05-22

**Authors:** Sebastian Arts, Martine van Bilsen, Erik J. van Lindert, Ronald HMA Bartels, Rene Aquarius, Hieronymus D. Boogaarts

**Affiliations:** Department of Neurosurgery, Radboud University Medical Center, 6525GA Nijmegen, The Netherlands; martine.vanbilsen@radboudumc.nl (M.v.B.); erik.vanlindert@radboudumc.nl (E.J.v.L.); ronald.bartels@radboudumc.nl (R.H.B.); rene.aquarius@radboudumc.nl (R.A.); Jeroen.Boogaarts@radboudumc.nl (H.D.B.)

**Keywords:** external cerebrospinal fluid drain, LiquoGuard^®^7, hydrocephalus

## Abstract

Background: Automated cerebrospinal fluid (CSF) drainage systems allow for the mobilization of patients with an external CSF drain. The aim of this study is to describe the implementation of an automated CSF drainage system in neurosurgical patients with external CSF drains. Methods: A feasibility study was performed using an automated CSF drainage system (LiquoGuard^®^7, Möller Medical GmbH, Fulda, Germany) in adult neurosurgical patients treated with external lumbar or external ventricular drains between December 2017 and June 2020. Limited mobilization was allowed—patients were allowed to adjust their inclined beds, sit in chairs and walk under the supervision of a nurse or physical therapist. The primary outcome was the number of prematurely terminated drainage sessions. Results: Twenty-three patients were included. Drainage was terminated prematurely in eight (35%) patients. In three (13%) of these patients, drainage was terminated due to signs of hydrocephalus. Pressure-controlled drainage in patients with external lumbar drains (ELD) showed inaccurate pressure curves, which was solved by using volume-controlled drainage in ELD patients. Conclusion: The implementation of an automated CSF drainage system (LiquoGuard^®^7) for CSF drainage allows for early mobilization in a subset of patients with external CSF drains. External lumbar drains require volume-based drainage rather than differential pressure-dependent drainage.

## 1. Introduction

Temporary treatment of hydrocephalus using external lumbar or ventricular drainage is common in neurosurgical practice. Conventional hydrostatic pressure-controlled drainage with dripping chambers are the gold standard [1]. However, using dripping chambers restricts patient mobility and can cause unintentional over- or underdrainage during Valsalva manoeuvres or patient movement. Patients are therefore immobilized during drainage.

It has been shown that early mobilization decreases the complications associated with immobilization, results in a shorter length of hospital stay and seems to be safe for patients with an external cerebrospinal fluid (CSF) drain [2,3,4,5,6,7]. To prevent over- and underdrainage, external drains are clamped during mobilization and require continuous intracranial pressure monitoring (ICP), which limits mobilization time [5,6,7].

The LiquoGuard^®^7 (Möller Medical GmbH, Fulda, Germany), is an automated cerebrospinal fluid (CSF) drainage system that has been available since 2011. It acts as a self-adjusting, pressure-based resistor that allows CSF to drain in a controlled manner, and is thus intended to prevent over- or underdrainage [1]. The system is designed to enable early mobilization of patients. Its use is described in patients requiring CSF drainage due to thoracoabdominal aortic aneurysm repair and in neurosurgical patients requiring ICP pressure regulation [1,8,9,10]. However, to our knowledge, its capacity to facilitate the mobilization of neurosurgical patients has not been previously described.

The aim of this study is to describe the implementation of the LiquoGuard^®^7 in mobilized hydrocephalus patients with external CSF drains.

## 2. Materials and Methods

This feasibility study was performed in accordance with the ethical standards of the institutional board review (NL62883.091.17). From December 2017 until June 2020, adult neurosurgical patients (>18 years) treated with an external ventricular drain (EVD, CODMAN^®^ BACTISEAL^®^ EVD Catheter, Johnson & Johnson, Raynham, MA, USA) or an external lumbar drain (ELD, Perifix^®^ epidural catheter, B Braun, Melsungen AG, Germany) were included.

**Figure 1:**
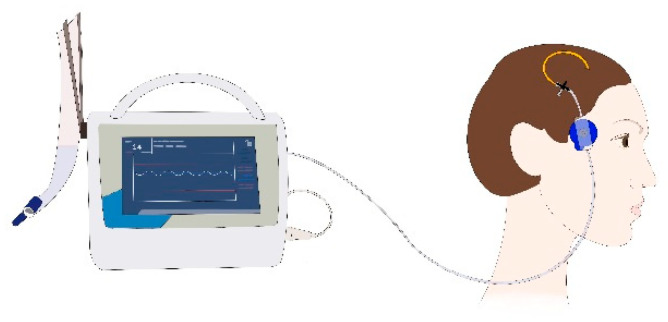
Set-up of the LiquoGuard^®^7 in a patient treated with an EVD. The pressure sensor is attached to the skin by an electrocardiogram sticker at the height of the foramen of Monro.

Two LiquoGuard^®^7 systems (Möller Medical GmbH, Fulda, Germany) were used. The device is an automated pressure-based CSF drainage system connected to the EVD or ELD catheter. It measures ICP by using a pressure sensor that is fixed by an electrocardiogram sticker on the skin over the temporal bone at the height of the foramen of Monro (Figure 1) [9].

The manufacturer gave instruction on the theoretical background of the device and its practical use to all caregivers. The first author (S.A.) and a nurse practitioner were available to answer remaining questions during the implementation and to provide assistance to daily caregivers. User manuals, in the form of flowcharts, were also attached to each system.

The placement of the EVDs and ELDs was performed according to the institutional standard insertion protocol. At the intensive care unit (ICU), patients were treated with a conventional drainage system (Duet™ External Drainage and Monitoring System, Medtronic, Minneapolis, MN, USA). After transfer to the neurosurgical ward, the LiquoGuard^®^7 was installed if written and verbal informed consent was obtained from patients, or their legally authorised representatives. Patients were included in the study on a first-come, first-served basis, due to restricted availability of the system.

Two drainage modes are available for the LiquoGuard^®^7—pressure-regulated drainage and volume-regulated drainage. In this study, pressure-regulated drainage was used.

In pressure-regulated drainage, the system drains CSF as soon as intracranial pressure exceeds the pre-set pressure value. The device produces an audible alarm when pre-set alarm limits are exceeded. Our conventional system is calibrated at 10 cm above the foramen of Monro per institutional protocol. Accordingly, the used pre-set pressure value for the LiquoGuard^®^7 was set to 10 cmH2O, with an alarm range between −10 cmH2O and 20 cmH2O. In volume-regulated drainage, the system drains a pre-set volume of CSF per hour, regardless of intracranial pressure. According to the instructions for use, the diameter of the lumen of the EVD and ELD catheters used needed to be more than 0.7 mm in order to measure CSF pressure correctly.

Mobilization was encouraged as much as was clinically possible. Patients received physical therapy once a day and were seated for at least thirty minutes, three times a day. Furthermore, patients were allowed to adjust their inclined bed and walk under the supervision of a nurse.

The primary outcome was the number of prematurely terminated drainage sessions with the LiquoGuard^®^7. Reasons for termination were described. Complications were included as secondary outcome measures and were registered if they occurred between the placement of the first drain and 30 days after removal of the last drain.

Complications were defined according to our earlier study regarding complications of external CSF drainage in aneurysmal subarachnoid haemorrhage (aSAH) [11], and were divided into direct and indirect complications. Direct complications were defined as complications with a direct relation to the external CSF drain (i.e., drain dislodgement, drain occlusion and meningitis). Indirect complications were defined as complications that could not be directly related to the external CSF drainage. These indirect medical complications were divided into four subgroups: infection, delirium, pressure injuries and thromboembolic complications. The number of internal shunts after external drain placement was also registered. Thromboembolic processes were subdivided into deep-vein thrombosis and pulmonary embolisms. Deep-vein thrombosis consisted of a confirmed diagnosis by echo-Doppler, while for pulmonary embolisms a confirmed diagnosis by spiral-CT scan was required. Delirium was registered as a complication when patients had clinical signs of delirium in accordance with the Delirium Observation Scale, for which haloperidol was given [12]. Pressure injuries were defined by the pressure injury grading score as stated by the National Pressure Ulcer Advisory Panel in 2016 [13]. Infections were only registered as complications if antibiotic treatment was started. An infection was detected by monitoring the clinical condition of the patient combined with a rising C-reactive protein and leukocyte count or positive cultures. The EVDs or ELDs were considered to be dislodged when the drains were inadvertently partially or entirely removed. Occlusion was registered as a complication if drain reimplantation was needed.

Patient-specific data were retrieved from the digital patient information system (Epic Systems Corporation (2014), Madison, WI, USA). Information regarding demographics, drainage period, drain type, length of hospital stay, destination after discharge and complications were prospectively registered.

All data were analysed using IBM SPSS Statistics for Windows (Version 22.0. IBM Corp., Armonk, NY, USA). Continuous data were presented as median and range. Categorical data were presented as counts and percentages.

## 3. Results

Twenty-three patients were included (Table 1). Nineteen EVDs and five ELDs were connected to the LiquoGuard^®^7 (one patient received an ELD after the EVD was removed). The median time from drain placement until mobilization (connection to the LiquoGuard^®^7) was three (0–20) days, with a median mobile period of six days (Table 1).

**Figure 2:**
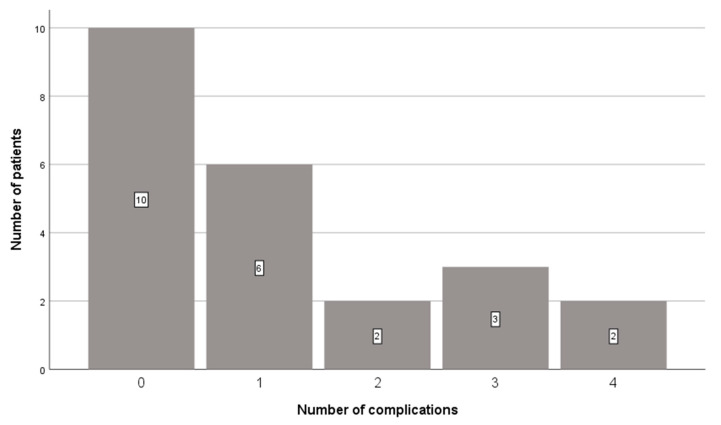
The number of complications per patient.

Twenty-eight complications occurred in 14 patients (61%). A total of nine direct complications and 19 indirect complications occurred in these patients, with a median of one (0–4) complication per patient (Figure 2, Table 2).

One drain dislodgement occurred while connected to the LiquoGuard^®^7. LiquoGuard^®^7′s tubing set occluded twice. In these cases, the LiquoGuard^®^7 was removed instead of being replaced.

The LiquoGuard^®^7 was disconnected prematurely in eight patients (35%) (Table 3). The first patient treated with the system became clinically unstable due to multi-organ failure and was reconnected to the conventional system. Any causal link with the device was excluded.

Although the lumen of the ELD tubing set used was more than 0.7 mm, it was observed that the pressure curves were not accurate in the first patient with an ELD. No pulsation was measured, and the pressures shown were constantly negative, even in supine position. As a result, the device did not drain the desired amount of CSF, which lead to underdrainage. After contacting a technical expert from the manufacturer, the drainage mode in patients treated with an ELD was set to volume-regulated with a pre-set volume of 10 mL/h, which solved the problem.

Despite pressure or volume measurements within limits and normal pulsations, three patients neurologically deteriorated while being drained with the LiquoGuard^®^7 (Table 4). All three patients showed clinical improvement after changing to a conventional drainage system.

## 4. Discussion

Neurological deterioration due to hydrocephalus occurred three times despite drainage systems being functional. This suggested that settings different from a normal overflow system, depending on hydrostatic pressure, might be required.

Patients with conventional external CSF drainage systems tend to moderately over-drain CSF, which occurs especially during manoeuvres that cause a short increase in intracranial pressure, for example coughing, leg elevation, positional changes or speaking. This overdrainage is prevented by the LiquoGuard^®^7, which may contribute to the observed neurological deterioration in some patients. Measuring ICP exclusively is possibly insufficient for maintaining CSF homeostasis, due to normal pressure hydrocephalus in aSAH patients [14,15]. Since the majority of patients in this series suffered from aSAH, normal pressure hydrocephalus might have contributed to the relatively high number of premature disconnections.

It may be that standardized pressure settings in these patients are insufficient. To overcome this problem, lowering the pressure settings stepwise or switching to volume-controlled drainage may help to drain sufficient amounts of CSF.

Additionally, when patients are in an upright position the physiological intracranial pressure can become negative, varying with exercise or movement [16,17]. This may result in underdrainage during mobilization, as pressure settings remain unchanged during mobilization. One would expect this relative underdrainage to be corrected during bed rest. However, this correction did not occur. This supports the idea that the regulation of CSF dynamics is complex and cannot always be limited exclusively to pressure or flow control in a mobilized patient. Continuous flow may be very important for maintaining an equilibrium in CSF homeostasis.

Although inaccurate pressure curves are a well-known phenomenon, the reason for inaccurate pressure curves in patients treated with an ELD is unknown. It is remarkable that previous literature regarding thoracic surgery shows adequate pressure measurements in lumbar drainage [8,10]. Although not described, it may be the case that, in these studies, pressure sensors were attached near the ELD insertion place. Since patients were mobilized in our study, the pressure sensor was attached at the height of the foramen of Monro, so pressure waves might not have been conducted well through the longer catheter. A second possible explanation is that intracranial haemorrhage compartmentalization of CSF may occur, which results in a discrepancy between measured spinal CSF pressure and intracranial CSF pressure.

Previous studies describe that the LiquoGuard^®^7 shows inappropriate curves and drains an inadequate amount of fluid from patients with slit ventricles [1,9]. In this study, no patients suffered from slit ventricles and, therefore, no experience was gained on this potential problem.

It is unclear which patient will benefit the most from automated CSF drainage with the LiquoGuard^®^7. Pressure and volume settings seem patient specific and underlying factors are currently unknown, which hampers patient selection for automated CSF drainage.

The incidence of meningitis is relatively high and not related to drainage time [18]; however, due to the small number of patients, no conclusions can be drawn concerning the number of complications. However, we have reported on all types of complications for transparency reasons. Theoretically, using the LiquoGuard^®^7 could lead to a decline in complications since mobilized patients might be less prone to secondary complications [2,3,5,6,7].

While shunt dependency in this study seemed slightly higher in comparison to our earlier series regarding complications in patients treated with a conventional EVD or ELD system (22% in this study versus 15%) [11], future studies should compare patients that receive automated CSF drainage and drainage with a conventional system. The sample size of the present study is small; however, the presented results are beneficial for other neurosurgical departments planning to implement the LiquoGuard^®^7 in their clinical practise.

## 5. Conclusions

The implementation of an automated CSF drainage system (LiquoGuard^®^7) for CSF drainage allows early mobilization in a subset of patients with an external CSF drain. External lumbar drains require a volume-based drainage rather than a differential pressure-dependent drainage.

## Figures and Tables

**Table 1:** Demographics data are presented as counts or median and range.

		Total (n)
Number of Patients		23
Gender (M:F) *		9:14
Age		59 (26–76)
Diagnosis	SAH	15 (65%)
	Tumour	1 (4.3%)
	ICH *	5 (22%)
	Ventriculitis After VPD Placement	2 (8.7)
Type of Drain	EVD	17
	ELD	4
	Both	2
Number of Drains		1 (1–2)
Total Drainage Period (days)		13 (7–41)
Drainage Period LG (days)		6.0 (1–16)
Drainage Period before Connected to LG (days)		3.0 (0–20)
Length of Stay (days)		18 (12–45)
ASA *		2 (1–3)
VPD *		5

* M: male; F: Female; ICH: intracerebral haemorrhage; LG: LiquoGuard^®^7; ASA: American Society of Anaesthesiology score; VPD: ventriculoperitoneal drain.

**Table 2:** Complications.

Complication	Group	Counts (%)
Direct (Drain-Related)	Meningitis	5 (13%)
	Dislodgement	3 (7.9%)
	Occlusion	1 (2.6%)
Indirect (Medical)	Infection	10 (26%)
	Delirium	2 (5.3%)
	Pressure Injuries	5 (13%)
	Thromboembolic Process	2 (5.3%)
	Total	28 (100%)

**Table 3:** Reasons for LiquoGuard^®^7 removal.

	Number	Percentage
Successful Challenge	13	57%
Clinically Instable	1	4.3%
Technical Problems	1	4.3%
Signs of Hydrocephalus	3	13%
Distal Occlusion	2	8.7%
Drain Dislodgement	1	4.3%
Other *	2	8.7%

* Other consists of one VPD insertion and one discharge to another department.

**Table 4:** The characteristics of patients that showed signs of hydrocephalus during drainage with the LiquoGuard^®^7.

Patient	Age	Diagnose	WFNS	EVD/ELD	Number of Drains	DP *	DP * LG *	Symptoms	Number of Complications
1	65	aSAH	3	EVD	1	19	6	Bradyphrenia, apraxia and ventriculomegaly	2
2	61	aSAH	1	ELD	2	19	16	Headache and CSF leakage along drain	3
3	72	PMH *	N/A	ELD	1	8	1	Headache	0

* DP: drainage period; LG: LiquoGuard^®^7; PMH: perimesencephalic haemorrhage.

## Data Availability

Data can be requested by sending an email to the corresponding author.
